# Saharan dust inputs and high UVR levels jointly alter the metabolic balance of marine oligotrophic ecosystems

**DOI:** 10.1038/srep35892

**Published:** 2016-10-24

**Authors:** Marco J. Cabrerizo, Juan Manuel Medina-Sánchez, Juan Manuel González-Olalla, Manuel Villar-Argaiz, Presentación Carrillo

**Affiliations:** 1 Departamento de Ecología, Facultad de Ciencias, Universidad de Granada, Campus Fuentenueva s/n, 18071, Granada España; 2 Instituto Universitario de Investigación del Agua, Universidad de Granada, C/ Ramón y Cajal, 4, 18071, Granada España

## Abstract

The metabolic balance of the most extensive bioma on the Earth is a controversial topic of the global-change research. High ultraviolet radiation (UVR) levels by the shoaling of upper mixed layers and increasing atmospheric dust deposition from arid regions may unpredictably alter the metabolic state of marine oligotrophic ecosystems. We performed an observational study across the south-western (SW) Mediterranean Sea to assess the planktonic metabolic balance and a microcosm experiment in two contrasting areas, heterotrophic nearshore and autotrophic open sea, to test whether a combined UVR × dust impact could alter their metabolic balance at mid-term scales. We show that the metabolic state of oligotrophic areas geographically varies and that the joint impact of UVR and dust inputs prompted a strong change towards autotrophic metabolism. We propose that this metabolic response could be accentuated with the global change as remote-sensing evidence shows increasing intensities, frequencies and number of dust events together with variations in the surface UVR fluxes on SW Mediterranean Sea. Overall, these findings suggest that the enhancement of the net carbon budget under a combined UVR and dust inputs impact could contribute to boost the biological pump, reinforcing the role of the oligotrophic marine ecosystems as CO_2_ sinks.

Several global studies during the last two decades have shown the high prevalence of heterotrophic metabolism into the ocean, particularly in oligotrophic ocean areas^1^,^2^. Currently, however, the metabolic balance of oceans poses a heated debate, as reflected in the studies of Williams *et al.*^3^ and Duarte *et al.*^4^ where their autotrophy or heterotrophy, respectively, are defended. Nevertheless, both perspectives agree that the oligotrophic ocean is a ‘single steady-stable’ ecosystem where the metabolic balance remains roughly invariant across spatio-temporal scales. By contrast, Serret *et al.*^5^ question the paradigm of the heterotrophy and propose that the oligotrophic ocean is “neither auto- nor heterotrophic, but functionally diverse”. Most of the results published to date on this issue derive from oceanographic transects and short-term (hours) experiments. Moreover, most of these studies have involved almost exclusively open-ocean areas, whereas less (or scarce) attention has been directed towards coastal areas, where previous studies have shown a contrasting response of planktonic metabolism (autotrophy or heterotrophy)^6^.

Global climate change is expanding the stratification in nearshore and open-sea oligotrophic areas (i.e. oceanic gyres)^7^ and increasing faster respiration rates than photosynthesis, as the Metabolic Theory of Ecology predicts^8^ and as observations have confirmed^9^. Global-change research also predicts higher climate variability, with greater nutrient availability due to severe droughts and harsher aridity, and increases in solar radiation exposure due to shallower upper mixed layers^10^. Deserts supply prodigious amounts of dust that affect both human concerns (i.e., weather, climate and health)^11^,^12^ and ecosystem productivity by providing key nutrients to stimulate the growth of planktonic communities^13^,^14^. Particularly in aquatic ecosystems, studies have reported that nutrients associated with dust inputs tend to stimulate primary production (PP)^15^,^16^,^17^ and bacterial production^18^, altering the structure of microbial planktonic communities^19^,^20^ or biogeochemical cycles^21^. Other studies, however, have also shown negative effects of dust on marine biota, due to the presence of high concentrations of toxic elements for growth (e.g. copper, cadmium or lead)^22^.

Together with dust-derived nutrient inputs, the other key factor that modulates the plankton responses is solar radiation. Within the euphotic zone, autotrophic organisms use photosynthetically active radiation (PAR) to drive photosynthesis, but in the upper part of this layer, cells are also exposed to high levels of ultraviolet radiation (UVR). A huge body of literature has shown their negative effects on metabolism and physiology of autotrophs^23^ and heterotrophs^24^. However, positive effects of UVR on both compartments have also been shown, such as increased photorepair DNA damage^25^,^26^ or enhanced photosynthesis^27^.

Thus, understand the impact of these stressors (dust deposition and UVR) on planktonic communities is central for predicting the effects of global change on the metabolic balance, and consequently, determine whether planktonic communities act as net sinks or sources of CO_2_ affecting the atmosphere-ocean transfer^28^. In fact, a growing body of literature shows that the stressors can interact suppressing or amplifying their effects; hence their impacts can differ from their additive or single effects leading to ecological surprises^29^,^30^. However, currently there is no direct empirical evidence quantifying how the effects of dust inputs and UVR on the planktonic metabolic balance shifts across temporal and spatial scales in environments with heterogeneous physical, chemical and biological characteristics^31^,^32^, such as nearshore and open-sea areas. Comparatively, nearshore areas usually have higher concentrations of organic and inorganic materials than does the open sea due to the great influence of riverine and continental runoff inputs, which also diminish the penetration of UVR into the water column^33^. Thus, nearshore plankton communities are commonly more sensitive to UVR impact than are those of the open sea as the cells are adapted to darker environments and possess fewer protective mechanisms^34^.

With this background, in this work we quantified for the first time (i) total PP (PP_total_) and community respiration (CR) rates and the production/respiration metabolic balance (P/R ratio) across the south-western Mediterranean Sea (Alboran Sea); and (ii) the combined impact of Saharan dust inputs under high UVR levels on metabolic balance of planktonic communities through a microcosm experiment from two contrasting areas, one heterotrophic and other autotrophic. Ultimately, our aim was to identify whether the interaction of the two stressors will change the metabolic balance in these oceanic areas over short and mid-term scales in a future global-change scenario.

## Results

### Long-term trends in Aerosol index (AI) and short-wave (SW) fluxes on SW Mediterranean Sea

**Figure 1:**
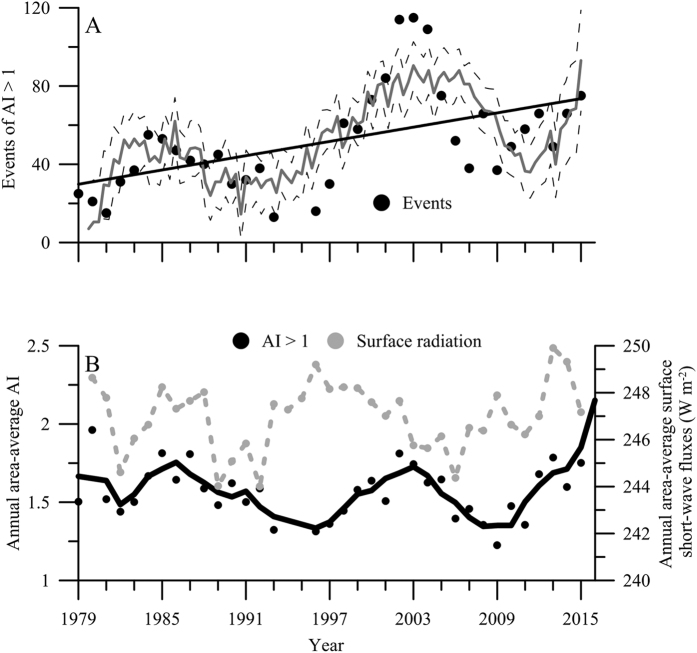
(**A**) Temporal pattern of total events of area-average aerosol index (AI > 1) for the 1979–2016 period. Solid grey trend line denotes the temporal response pattern using a non-linear fit of annual AI > 1 events *vs.* time with the Levenberg-Marquardt model. Dashed lines represent 95% confidence bands and the solid black line the regression line. (**B**) Mean annual area-average aerosol index (black circles, AI > 1, relative units) and surface short-wave radiation fluxes (grey circles, in W m^−2^) on Alboran Sea for 1979–2016 period. Solid lines represent smoothed trend for both measurements using a polynomial Savitzky-Golay fitting model.

The daily area-average AI and monthly area-average surface SW fluxes varied greatly through the 1979 and 1980–2016 period (Fig. S1). This variability resulted in alternating years with a characteristically strong seasonality in aerosol inputs (i.e. increasing AI values from May to September and maximum peaks in July-August) (e.g. year 2012, Fig. S1) followed by years where a slightly constant input of aerosols occurred (e.g. year 2013–2014, Fig. S1). AI also showed a clear oscillation in the total number of events, as well as, in the annual values over the study period (Fig. 1). Thus, we found periods of 6–8 years where total number of AI > 1 events significantly increased or decreased (Fig. 1). Despite these oscillating periodic cycles, a significantly increasing frequency in the events registered was found over a long-term scale (Fig. 1; R^2^ = 0.28, F_12.51_, p < 0.01). For SW fluxes, a marked intra-annual seasonality was found (Fig. S1); however, these fluxes did not exhibit a clear trend over the time (Fig. 1).

### Observational study

#### Metabolic balance

In relation to the underwater radiation field, the profiles in all stations showed a high transparency to UVR (*kd*_305_ = 0.32–0.44, *kd*_320_ = 0.23–0.77, *kd*_380_ = 0.1–0.93) and PAR (*kd*_PAR_ = ranging between 0.01–0.14). Seawater temperature was relatively uniform across the SW Mediterranean Sea with mean values ~21.47 °C (±0.71), from surface to 25 m deep across the study section (Table S1). Chlorophyll *a* (Chl *a*) concentrations were very low, with values below 1 μg L^−1^, excepting four cases (stations 3, 5, 6 and 7), where chl *a* concentrations rose to ~2 μg L^−1^ (Table S1).

Nutrient concentrations were also generally low (Table S1), particularly total phosphorus (TP) with values <0.9 μM, whereas total nitrogen (TN) showed values ranging between 228 and 357 μM across the Mediterranean Sea section studied. Dissolved organic carbon (DOC) concentrations averaged 197 μM, although with concentrations varying between ~119 and 323 μM (Table S1).

**Figure 2:**
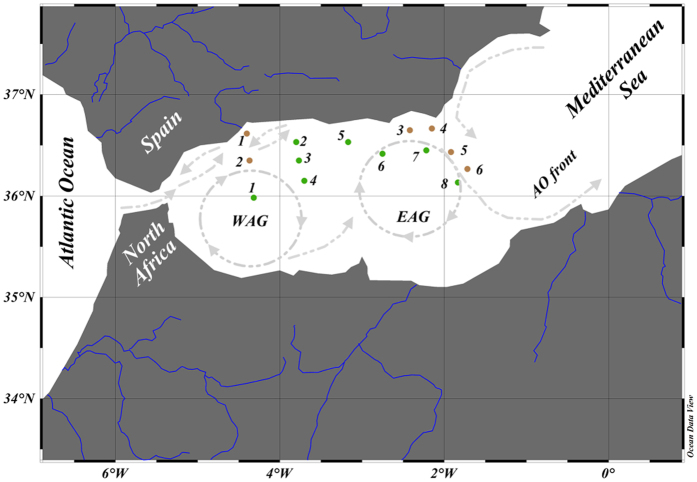
Geographical distribution of the different stations where the planktonic metabolism was measured during the Microsens campaign. Brown and green circles represent heterotrophic and autotrophic stations, respectively, whereas uppercase abbreviations means Western anticyclonic (WAG), Eastern anticyclonic (EAG) gyres and Almeria-Oman front (AO) and blue lines the main rivers in southern Spain and North Africa. Map was created using Ocean Data View v. 4.7.6 (https://odv.awi.de). Note that gyres are represented in relative magnitude and shape.

PP_total_ varied between 10 (±1) and 118 (±1) mmol C m^−3^ d^−1^, whereas, CR did between 33 (±12) and 105 (±0.10) mmol C m^−3^ d^−1^. This range of values in PP_total_ and CR rates was translated into P/R ratios that ranged between 0.15 and 1.84, indicating an alternation between auto and heterotrophic stations across the studied section (Fig. 2; Table S1).

### Experimental study

#### Physical, chemical and biological conditions

Sky conditions during the experimental period were characterized by slightly overcast days, particularly on June, 19^th^ and 20^th^. Maximum irradiance received by samples during the experiment reached 6.4 (for 305 nm), 35.1 (for 320 nm) and 93.6 (for 380 nm) μW cm^−2^ and 534.9 (for PAR) W m^−2^, whereas mean daily irradiance during the exposure period oscillated between 2.03–2.82, 15.60–19.30 and 41.60–52.30 μW cm^−2^ and 225.60–282.30 W m^−2^, respectively, for 305, 320, 380 nm, and PAR (Fig. S2).

At the beginning of the experiments, both areas showed high TN and low TP concentrations, and high sestonic N:P ratios which were 2-fold higher in heterotrophic than in autotrophic communities (Table S2). By contrast, DOC concentrations were similar in both areas at the beginning (*t*-test = 1.45, p > 0.05) and overall decreased through the experiments, up to mean concentrations of 188.65 (±2.26) and 196.68 (±9.43) μM for heterotrophic and autotrophic communities, respectively.

**Figure 3:**
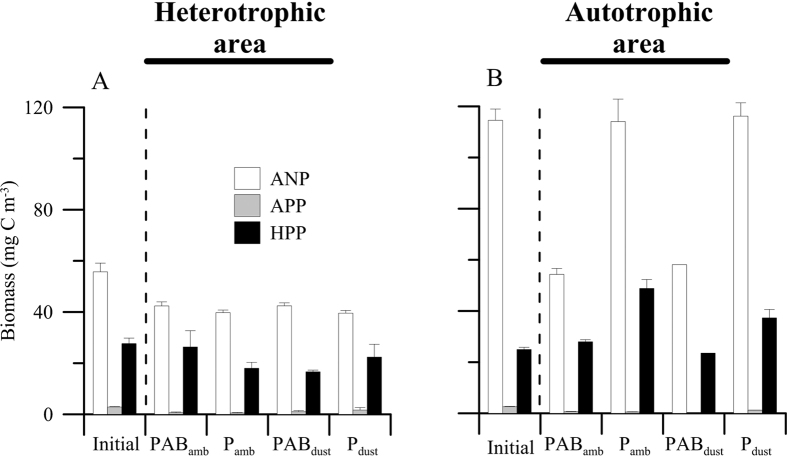
Mean (±SD) biomass (in mg C m^−3^) (**A,B**) of autotrophic nanoplankton (ANP), autotrophic picoplankton (APP) and heterotrophic picoplankton (HPP) in the microcosms at the beginning of the experiment (initial) and after 5 days of exposure under PAB (>280 nm) and PAR (P > 400 nm) radiation treatments and two dust treatments, ambient (amb) and dust treatments in the heterotrophic and autotrophic area.

In terms of biomass, the autotrophic nanoplankton (ANP) fraction dominated the community in both areas and in all treatments with respect to the heterotrophic picoplankton (HPP) (<40 μg C L^−1^) and autotrophic picoplankton (APP) fraction (<2 μg C L^−1^) over the experiment. However, only in open sea did UVR negatively affect the ANP and HPP fractions regardless the dust treatment, significantly lowering values by ca. 50% (Fig. 3; Table S3).

#### UVR and Dust effects on the metabolic balance

**Figure 4:**
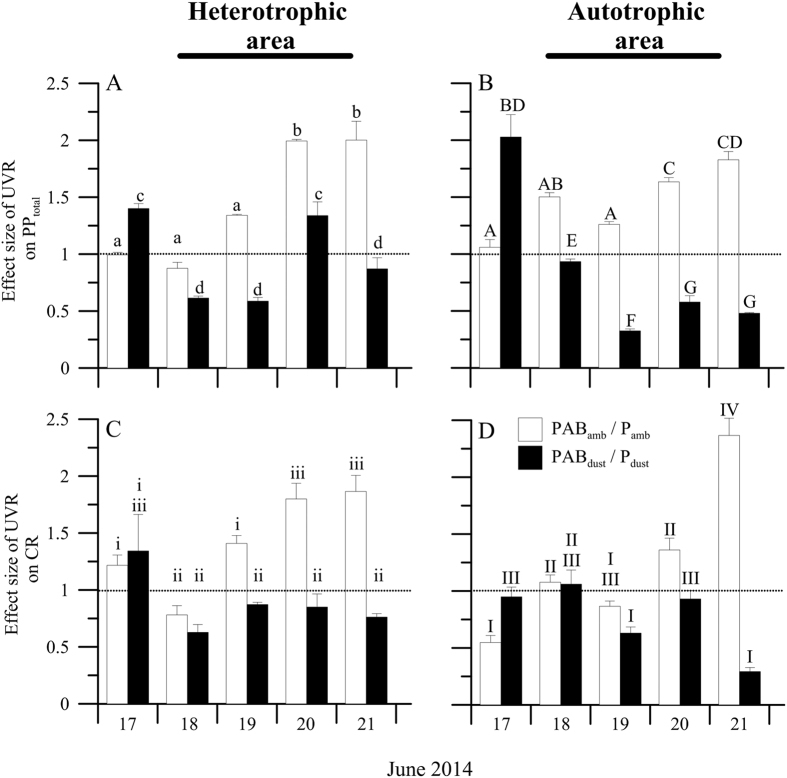
Effect size of UVR (as PAB/PAR ratio) on the mean (±SD) total primary production (**A,B**) (PP_total_, in mmol C m^−3^ d^−1^) and (**C,D**) mean (±SD) community respiration (CR) rates (in mmol C m^−3^ d^−1^) in the microcosms during the experiment under two dust treatments, ambient (amb) and dust, in the heterotrophic and autotrophic area. The letters on top of the bars indicate the result of the LSD *post hoc* test, and the horizontal dashed line delineates the positive (>1) or negative (<1) ultraviolet radiation effects.

In the heterotrophic area, PP_total_ significantly increased in all treatments, with values ranging between ~10 at the beginning and up to ~55–60 mmol C m^−3^ d^−1^ at the end of experiment (Fig. S3; Table S4). In the autotrophic area, PP_total_ slightly increased only under PAB_amb_ and P_dust_ treatments with respect to the initial conditions (Fig. S3) but significantly decreased under a combined impact of UVR × Dust. In both areas, the effect size of UVR was stimulatory on PP_total_ under ambient nutrient conditions, although dust addition significantly altered this stimulatory effect of UVR on PP_total_ over the experiment, generating an inhibitory effect that ranged between 30–57% and 38–74%, for the heterotrophic and autotrophic area, respectively (Fig. 4).

By contrast, CR showed significantly higher values at the beginning (heterotrophic area, ca. 28–40 mmol C m^−3^ d^−1^ and autotrophic area, 30–70 mmol C m^−3^ d^−1^), which declined over the experiment up to similar rates in both areas regardless of the treatment considered (except PAB_amb_ in autotrophic area) (Fig. S3; Table S4). As with PP_total_, UVR increased the CR in both areas, whereas the dust also significantly altered the effect size of UVR on CR, increasing the inhibition of CR by between ~20–60% and 2–88% in the heterotrophic and autotrophic area, respectively (Fig. 4; Table S4).

**Figure 5:**
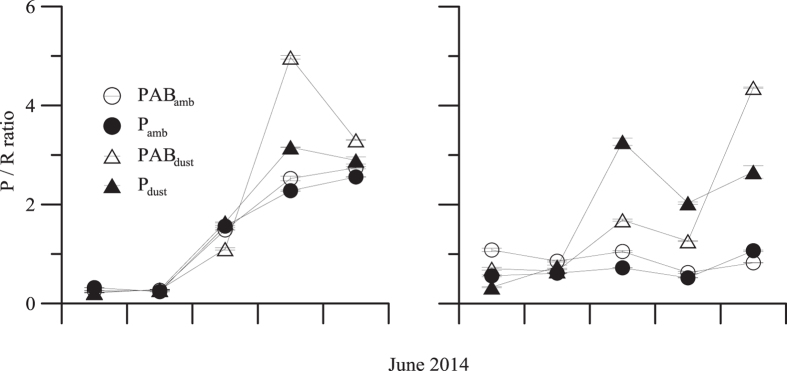
Mean (±SD) total production/respiration (P/R) ratios (**A,B**) under two radiation treatments, PAB (>280 nm) and PAR (P > 400 nm) and two nutrient treatments, ambient (amb) and dust during the experiments in the heterotrophic and autotrophic area.

From PP_total_ and CR data (Fig. S3) we calculated the P/R ratio during the experiments, which show a similar response pattern in both areas (Fig. 5). Thus, at short-term P/R ratio was <1 in both areas and for all treatments (with the exception of PAB_amb_ in autotrophic area, P/R = 1.09), indicating a clear heterotrophy. Conversely, from the third day to the end of the experiment the P/R ratio was >1, particularly under PAB_dust_ treatments, due to a surge in PP_total_ coupled with a fall in the CR rates. However, P/R ratio was ~1 or consistently <1 in the autotrophic compared to the heterotrophic area (P/R > 1) under ambient conditions, due to higher CR than PP_total_, as consequence of increased bacterial respiration under these conditions (data not shown). The metabolic balance changed from heterotrophy (or steady stable communities, P/R ~1) towards strong autotrophy at the end of the experiment under the joint effect of UVR and dust treatments (values between 3.3–4.4 for the heterotrophic and autotrophic area, respectively).

## Discussion

In this study, we report that the metabolic balance of strongly P-limited oligotrophic marine areas vary geographically between heterotrophic and autotrophic states, challenging the mainstream view of oligotrophic ocean as a ‘single steady-state’ ecosystem with an invariant metabolism across spatio-temporal scales^3^,^4^. However, this spatial pattern showed is consistent with the recent results of Serret *et al.*^5^ who showed that the Atlantic Ocean (i.e. Oceanic gyres) is neither autotrophic nor heterotrophic but metabolically diverse.

In addition, the values of the metabolic balance of the SW Mediterranean Sea are also in line with previous estimates for other oligotrophic areas worldwide, such as NW and East basin of the Mediterranean Sea (mean P/R = 0.74), the North (mean P/R = 0.75) and South Pacific Ocean (mean P/R = 1.17) and the Indian Ocean (mean P/R = 1.78)^6^. However, our findings in unproductive waters were between 4 to 7-fold lower that those reported for strongly productive areas in high-latitude environments, i.e. Southern Ocean and Arctic waters. The differences between both areas may be due not only to higher nutrients availability but also the fact that most of studies in the latter areas have been performed during the boreal/austral spring or summer in absence of a dark period in these latitudes. One underlying mechanism that likely can explain these contrasting results may be a strong stimulation of the bacterial respiration and dark-repair processes^35^ during the night at temperate latitudes. However, these processes are inhibited under the midnight sun in the spring-summer Arctic day as recent studies have reported^36^,^37^.

In view of the metabolic variability observed in our survey and in previous reports over the last two decades^6^ we investigated whether the metabolic state from two contrasting areas, one heterotrophic and the other autotrophic, could be altered by the joint action of two major environmental stressors, i.e. Saharan dust inputs and UVR, in a future global-change scenario. Our data further suggests that the trophic state of both areas at the beginning of the experiments was most likely associated with plankton physiological conditions. Thus, not only Chl *a* concentrations were lower, but also the P-limitation was higher and the sestonic C:P and N:P ratios were 2-fold higher in the heterotrophic than the autotrophic area.

We found an inhibitory effect of the two stressors acting together on PP_total_. These findings partially agree with previous studies in oligotrophic ecosystems (fresh and marine) that showed a higher negative UVR effect after nutrient enrichment on PP due to a decoupling between photosynthesis and growth^38^,^39^. Likewise, a negative synergistic interactive effect of UVR and dust on CR was found. This finding contrasts with recent results showing a rise in respiratory processes under UVR due to an enhanced heterotrophic metabolism^36^. By contrast, although dust addition inverted the stimulatory UVR effect, we found that the planktonic metabolic balance showed a clear response pattern towards autotrophy in SW Mediterranean Sea communities at the mid-term, regardless of the area considered. Therefore, greater dust pulses due to a higher frequency and intensity of dust export events from Sahara, as reported here (see Fig. 1), together with high UVR fluxes by shallower upper mixed layers (UML) owing to increased surface temperatures, may induce significant changes in the metabolic state of oligotrophic marine ecosystems.

Despite that the UVR and dust inputs acting in concert depressed the respiratory processes, we found no biomass accumulation in any area over the experiment. The absence of stimulation in the autotrophic biomass compartment agrees with previous results that showed a very small biomass response after similar inorganic nutrient or dust additions^40^,^41^. The most believable explanation behind this observation could be a high lysis rates undergone by phytoplankton during this period, late spring/early summer, as previously shown by Agustí & Duarte^42^ in Mediterranean coastal waters. Nevertheless, based on our results we can rule out the idea that the stability found in phytoplankton biomass was due to: (i) a potential to toxic effect of metals contained in dust inputs as recent studies have proposed^43^, as in our study those concentrations were below detection limits (González-Olalla *et al.*, submitted); or (ii) competition with HPP by nutrients as we found no stimulation of this compartment, either, over the experiment in terms of biomass.

The trend towards an autotrophic balance reported through our experimental study suggests that the combined impact of UVR and dust would bolster the biological pump. Thus, we can speculate that the fate of a larger fraction of carbon fixed by phytoplankton could not be remineralization and release as CO_2_ into the atmosphere, as several previous works have suggested for unproductive waters^44^,^45^ but rather it could be exportation from the euphotic zone, contributing the biological pump and reinforcing the marine ecosystems as CO_2_ sinks^46^. However, we also should consider the potential role that mesozooplankton grazing and respiration play to remove marine primary production and increase carbon losses, respectively, as we directly excluded mesozooplankton in our study. If we assumed that mesozooplankton potentially consume ca. 23% of the total PP^47^,^48^ and the respiratory losses of mesozooplankton represent on average the 25% of C ingested from PP^49^, zooplankton feeding activity would not substantially alter the trend reported (see Fig. S4).

Finally, and although the metabolic balance in these areas continues to be a great challenge for oceanographic research, observational studies combined with mid-term multi-stressors experiments in contrasting marine areas constitute useful approaches to inform how future alterations due to the global climate change could alter the biotic regulation of CO_2_. Despite that we should be cautious in extrapolating these results from a regional scale to a global oceanic scale, they underscore the need to consider that these oligotrophic areas, which suppose about half of the Earth’s surface^5^ could potentially become carbon sinks, at least during certain periods of the annual cycle (e.g. high UVR fluxes, peaks dust inputs) in the upcoming future.

## Methods

### Remote-sensing study

Daily area-average aerosol index (AI) and surface SW fluxes (i.e., UVR) for all Alboran Sea region (36°43′ 5.1594″ – 35°22′ 0.4794″ N, 5°5′ 51.7194″ – 1°59′58.2″ W) (SW Mediterranean Sea) were downloaded from Giovanni v 4. 18. 3^50^. For 1979–2016 period, AI data used were provided by TOMS Nimbus-7 (January 1, 1979 – May 5, 1993), TOMS EP (July 22, 1996 – December 13, 2005) and OMI (December 14, 2005 – March 13, 2016) satellites. For 1980–2016, surface UVR-flux data used were provided by the MERRA-2 model. Previous studies in Mediterranean ecosystems have shown a highly positive correlation of AI with total phosphorus (TP) and particulate matter linked to dry atmospheric dust deposition^51^. We used annual means of area-average AI and SW-flux data as a measure of intensity of atmospheric dust deposition and incident irradiance on surface waters of Alboran Sea, respectively. Moreover, because previous studies in Mediterranean ecosystems have established that an AI = 1 constitutes the threshold between mild and intense events of dust deposition and AI > 1 are the main contributors to the total annual AI^52^, only AI > 1 were considered during the 1978–2015 period. We also estimated the annual frequency of these events as the number of days per year affected by intense deposition events.

### Observational and experimental study

Our study was conducted aboard of the Spanish B. O. Francisco de Paula Navarro cruiser from 16 to 21 June of 2014 during the MICROSENS campaign. The observational study comprised a total of 14 stations over the SW Mediterranean Sea to quantify the metabolism of planktonic communities. At each station, surface (5 m depth) seawater samples were collected using 10-L Niskin bottles and screened with 200 μm Nitex mesh to remove mesozooplankton (see all details about measurements below). In addition, additional integrated samples from the surface up to 15 m depth (300 L per area) in a nearshore (outside the West anticyclonic gyre, 36°37′ N, 4°24′ W - heterotrophic 1) and open-sea station (area inside this gyre, 35°59′ N, 4°19′ W – autotrophic 1) were also used in an experimental manipulation study, to assess the interactive effect of heavy Saharan dust inputs and high UVR fluxes, as expected in a global-change scenario (see details below). The rationale behind mixing water samples from various depths was to ensure a composite sample representative of the UML characterized by nearly uniform conditions. The sampling at discrete depths up to 15 m was also well justified by: (1) we did not found significant differences in P/R ratios between surface and at 15 m depth for both areas (data not shown) and (2) because a recent study in Alboran Sea during 1969–2012 have showed that during our sampling period the UML oscillate between 15 (±10)–30 (±15) meters^53^. In addition, we also considered previous findings by Marañón *et al.*^41^ and Pulido-Villena *et al.*^54^ that showed that a dust enrichment caused by a high deposition event can reach up to 10–15 m depth.

The original integrated seawater samples from each area were sorted, mixed and placed in two different opaque containers (150 L), one with seawater with ambient nutrient conditions and the other enriched with Saharan dust-nutrient inputs. Immediately after dust additions, the seawater was dispensed into microcosms consisting in 15-L UVR-transparent low-density polyethylene bags (LDPE) (Plasticos Andalucia, Spain). To assess interactive effects of UVR and Saharan dust inputs, a 2 × 2 matrix in triplicate was implemented with: (1) two radiation treatments, PAB (UVR + PAR > 280 nm, microcosms covered with LDPE) and P (PAR > 400 nm, microcosms covered with Ultraphan UV 395 Opak Difegra film) and (2) two dust treatments, ambient (amb) (nutrient conditions at the sampling moment) and dust (i.e. enriched with 4.1 mg L^−1^ final concentration of Saharan dust).

The LDPE used transmits 90% of photosynthetic active radiation, 75% of UV-A and 60% of UV-B, whereas Ultraphan UV 395 Opak Difegra film transmits >90% of PAR but screens out UVR (<390 nm). At the laboratory, and prior to the *in situ* experiment, the Saharan soil collected *in situ* in Merzouga (Tafilalet, Morocco; 31°06′.00 N 3°59′.24 W) was dry-sieved with a custom column knotted with wire mesh cloth of 1 mm and 100 mm pore size and dust was collected on a steel foil underneath the nest of sieves. The particles collected were then winnowed near a tilted glass and particles that adhered to the glass were gently collected with a fine brush. The size distributions of the sieved particles (1–10 μm) (Leitz Fluovert FS, Leica, Wetzlar, Germany) were broadly comparable with those of mineral dust collected in rain samples (~5–10 μm), although had a slightly smaller size (~10–20 μm) than sieved dust samples of Marañón *et al.*^41^. We used sieved dust samples within this range because of dust particles >20 μm are rapidly removed during the atmospheric transport; in addition, at a certain distance from its source, soil-derived dust constitutes a coarse aerosol mode with a mean range of particle size between 2–7 μm^55^.

The dust concentration simulated the dust enrichment caused by an event of high deposition (61.5 g m^−2^) in a 15-m surface-water layer. Recent observations show that annual dust-deposition rates vary widely (spatially and temporally) across the Mediterranean Sea, with values ranging between 2 to 27 g m^−2^ yr^−1^ in the western basin^56^. In addition, most of the Saharan dust-deposition events tend to occur in pulses, and hence sometimes a single event can account for ~40–80% of the annual flux registered into an ecosystem^57^. Therefore, in our experimental addition we simulate a plausible future deposition scenario which represents increases of more than 2-fold with respect to the total dust deposition received by the Mediterranean basin every year currently.

After the microcosms were filled and amended with dust, they were suspended in two large black-painted tanks (ca. 800 L each; 1 for nearshore samples and 1 for open-sea samples) where were incubated during five days. We maintained the *in situ* temperature in both tanks by continuously pumping surface sea water. In addition, we manually shook the microcosms every hour to avoid that organisms settle so that they would receive homogeneous irradiances. The samples used in the incubations were taken using a manual vacuum syringe connected to an acid-washed silicone tube inserted in each microcosm.

### Analyses and measurements

#### Solar radiation and physico-chemical characteristics of the water column

Incident solar radiation on microcosms was monitored daily using a BIC Compact multichannel radiometer (Biospherical Instruments Inc., CA, USA) installed on the top of the ship-deck with 3 channels for UVR portion, one for UV-B (305 nm) and two for UV-A 320 and 380 nm and one broadband channel for PAR (400–700 nm).

At each station, underwater profiles of solar radiation were made with a submergible-BIC Compact multichannel radiometer (Biospherical Instruments Inc., CA, USA) with the same 4 radiation channels that mentioned above. Vertical profiles of temperature, pH, salinity and conductivity (down to 25 m) were taken using a sealogger CTD SBE 25 (Sea-Bird Electronics, Inc., WA, USA).

#### Chlorophyll a (Chl a) measurements

At each station (3 L) and every day at sunrise from each microcosm (3 L on June 17^th^, and 1 L the following days), seawater samples were immediately filtered onto Whatmann GF/F filters (25 mm diameter) and stored at −20 °C until analysed. At the laboratory, the photosynthetic pigments were extracted in absolute methanol in darkness at 4 °C for 24 h^58^ and were measured using a fluorometer (Perkin Elmer, model LS 55) which is routinely calibrated using a chl *a* standard (chl *a* from spinach, Sigma-Aldrich).

#### Autotrophic plankton counting

During the first and the last day, water samples from each microcosm were taken and preserved in brown glass bottles (125 mL) using alkaline Lugol’s reagent (c. 1% vol/vol) to identify and quantify autotrophic nanoplankton (ANP). An aliquot of 50 mL from each sample was settled in an Utermöhl chamber of 2.6 cm diameter for 48 h to ensure complete sedimentation of the smallest phytoplankton species and counts were made at 400x and 1000x magnification under an inverted microscope (Carl Zeiss AX10, LCC, USA). Also, 1.5 mL of sample was fixed with 75 μL of particle-free 20% (w/v) paraformaldehyde (1% final concentration) and immediately frozen in liquid nitrogen until analysed to quantify cell abundance of autotrophic picoplankton (APP) using a flow cytometer (FACSCanto II, Becton Dickinson Biosciences, Oxford, UK) (see details below). For ANP samples, at least 400 cells of the most abundant species were counted, and 20 cells of each species were measured to estimate cell volume according to a corresponding geometrical shape^59^, whereas biovolume for APP was calculated following Ribés *et al.*^60^. Biovolumes were converted into carbon biomass using the formulas and coefficients proposed by Verity *et al.*^61^.

#### Heterotrophic plankton counting

Heterotrophic picoplankton (HPP) abundance was determined by the flow cytometry technique (FACSCanto II, Becton Dickinson Biosciences, Oxford, UK), fixing 1.5 mL of sampling water with 75 μL of particle-free 20% (w/v) paraformaldehyde (1% final concentration) and immediately frozen in liquid nitrogen until analysis. Before being analysed, unfrozen samples were stained using Syber Green I DNA (Sigma-Aldrich Co Ltd) 1:5000 final dilution^62^,^63^. In addition, yellow-green 1 μm beads at standard concentration (10^5^ particles mL^−1^) (Fluoresbrite Microparticles, Polysciences, PA, USA) were also added to determine the absolute cellular concentrations^62^,^64^. Phycoerythrin and chl *a* fluorescence signals were used to distinguish between APP and HPP^65^. HPP biomass values were estimated by approximating cell volume to their geometric shape and afterwards transform it to carbon units following suitable conversion factors^66^.

#### Nutrient and dissolved organic carbon analyses

Triplicate samples from each station and microcosm were placed in 300 mL PET plastic bottles and frozen (−20 °C) until nutrient analyses. To determine total nitrogen (TN), total dissolved nitrogen (TDN), total phosphorus (TP) and total dissolved phosphorus (TDP) concentrations, the samples were processed using the simultaneous persulphate oxidation method proposed by Koroleff 67.

For dissolved organic carbon (DOC) determination from each station and microcosm, triplicate seawater samples (150 mL) were filtered through pre-combusted (500 °C during 2 h) GF/F Whatmann filters (25 mm diameter). Afterwards, they were acidified with HCl 1N (2%) and stored in darkness at 4 °C until analysis. At the laboratory, DOC concentrations were measured with a TOC analyzer (Shimandzu, model 5000) following the procedure of Benner & Strom^68^.

#### ^14^Carbon incorporation measurements

One set of 42 samples in total for the observational study (duplicate clear bottles and one dark per station) plus one set of 32 daily samples for the experimental study (triplicate clear bottles and one dark bottle) were taken and inoculated with 5 μCi of labelled sodium bicarbonate to measure carbon incorporation as primary production (PP) following the method proposed by Steemann-Nielsen^69^. The experimental bottles were 35-mL UV-transparent Teflon FEP narrow-mouth bottles (Nalgene), uncovered (clear bottles) or covered with opaque adhesive foil (black bottles). All Teflon FEP bottles were then placed in the same tank of controlled-temperature as the microcosms and exposed for 4 h to solar radiation. Particulate PP was determined by filtering 30 mL through Whatmann GF/F filters (25 mm in diameter) at low pressure (<100 mmHg) to avoid cell breakage, whereas a subsample of 4 mL of the filtrate was directly collected in scintillation vials to assess ^14^C activity in the dissolved organic carbon fraction (i.e., DO^14^C). After this, filters and filtrates were exposed to acid fumes for 24 h by adding 1N HCl (2%) to eliminate the non-assimilated ^14^C. Finally, each sample was measured using a scintillation counter LS-6000 TA (Beckman). The total CO_2_ in seawater samples was calculated from the alkalinity and pH measurements^70^. In all calculations, the dark values were subtracted from the corresponding light values. PP_total_ was calculated as the sum of particulate PP plus the DOC fraction released by phytoplankton.

#### Oxygen concentration measurements

One set of 28 samples in total for the observational study (in duplicate per station) plus one set of 24 daily samples, for each experimental day (in triplicate per experimental treatment and area), were used to fill (without bubbles) 35-mL UV-transparent Teflon FEP narrow-mouth bottles (Nalgene) equipped with sensor spots (SP-PSt3-NAU-D5-YOP), sealed to avoid gas exchanges and incubated in darkness to measure the oxygen concentration during 12 h using an oxygen transmitter (Fibox 3, Presens GmbH, Germany) equipped with Oxyview 6.02 software to register data. Previously, the system was calibrated by a two-point calibration (0% and 100% oxygen saturation) together with temperature and atmospheric pressure data. From the resulting oxygen concentration data, we calculated the community respiration (CR) rates as the slope of the regression fit for decreases in oxygen concentration vs. time. The CR rates (in oxygen units) were converted into C units assuming a respiratory quotient of 1^71^.

#### Data and statistical analyses

We evaluated the effect size of UVR under both dust treatments on PP_total_ and CR as:


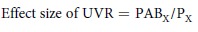


X being the ambient or dust treatment. A value > 1 mean a stimulatory ultraviolet radiation (UVR) effect, whereas <1 an inhibitory UVR effect for each nutrient treatment.

Statistical analyses for each experimental area (nearshore and open sea) were done separately. The effects of UVR, dust and their interaction on planktonic biomass of ANP, APP and HPP at the end of the experiment were tested by a two-way analysis of variance (ANOVA). Because several interactions occurred within each planktonic group, these are highlighted in the text accordingly. A one-way repeated measures ANOVA (RM-ANOVA) was used to test whether the dust addition modified the effect size of UVR on PP_total_ and CR over the experiment. When significant interactions were detected, a *post hoc* least significant differences (LSD) test was performed. Also, a linear regression analysis was applied to relate the trend in the number of events of AI > 1 vs. time. When appropriate, some of the data (e.g. DOC) were tested for significant differences using Student’s *t*-test. Data were checked for normality (Shapiro-Wilk’s test), and homoscedasticity (Levene’s test) assumptions. All data are reported as mean values and standard deviations, whereas error propagation was used in the effect size of UVR on PP_total_ and CR. The metabolic balance data for each station across the SW Mediterranean Sea were represented using Ocean Data View v. 4.7.6^72^.

## Additional Information

**How to cite this article**: Cabrerizo, M. J. *et al.* Saharan dust inputs and high UVR levels jointly alter the metabolic balance of marine oligotrophic ecosystems. *Sci. Rep.* **6**, 35892; doi: 10.1038/srep35892 (2016).

## Supplementary Material

Supplementary Information

## References

[b1] del Giorgio P. A., Cole J. J. & Cimbleris A. Respiration rates in bacteria exceed phytoplankton production in unproductive aquatic systems. Nature 385, 148–151 (1997).

[b2] Duarte C. M. & Agustí S. The CO_2_ balance of unproductive aquactic ecosystems. Science 281, 234–236 (1998). 9657712 10.1126/science.281.5374.234

[b3] Williams P. J. l. B., Quay P. D., Westberry T. K. & Behrenfeld M. J. The oligotrophic ocean is autotrophic? Ann.Rev. Mar. Sci. 5, 535–549 (2013). 10.1146/annurev-marine-121211-172335 22809190

[b4] Duarte C. M., Regaudie-de-Gioux A., Arrieta J. M., Delgado-Huertas A. & Agustí S. The oligotrophic ocean is heterotrophic. Ann. Rev. Mar. Sci. 5, 551–569 (2013). 10.1146/annurev-marine-121211-172337 22809189

[b5] Serret P. *et al.* Both respiration and photosynthesis determine the scaling of plankton metabolism in the oligotrophic ocean. Nat. Commun. 6, 6961 (2015). 25908109 10.1038/ncomms7961 PMC4462842

[b6] Regaudie-de-Gioux A. & Duarte C. M. Global patterns in oceanic planktonic metabolism. Limnol. Oceanogr. 58, 977–986 (2013).

[b7] Polovina J. J., Howell E. A. & Abecassis M. Ocean´s least productive waters are expanding. Geophys. Res. Lett. 35, L03618 (2008).

[b8] Brown J. P., Gillooly F., Allen A. P., Savage V. M. & West G. B. Toward a metabolic theory of ecology. Ecology 85, 1771–1789 (2004).

[b9] Yvon-Durocher G., Jones J. I., Trimmer M., Woodward G. & Montoya J. M. Warming alters the metabolic balance of ecosystems. Philos. T. Roy. Soc. B 365, 2117–2126 (2010). 10.1098/rstb.2010.0038 PMC2880133 20513719

[b10] IPCC. Climate Change 2013. The Physical Science Basis. 1–1535 (Cambridge University Press, New York, USA, 2013).

[b11] Vinoj V. *et al.* Short-term modulation of Indian summer monsoon rainfall by West Asian dust. Nat. Geosci. 7, 308–313 (2014).

[b12] Jickells T. D. *et al.* Global iron connections between desert dust, ocean biogeochemistry, and climate. Science 308, 67–71 (2005). 15802595 10.1126/science.1105959

[b13] Gunnarsson T. G., Arnalds O., Appleton G., Méndez V. & Gill J. A. Ecosystem recharge by volcanic dust drives broad-scale variation in bird abundance. Ecol. Evol. 5, 2386–2396 (2015). 26120428 10.1002/ece3.1523 PMC4475371

[b14] Moore C. M. *et al.* Processes and patterns of oceanic nutrient limitation. Nat. Geosci. 6, 701–710 (2013).

[b15] Ridame C. *et al.* Contrasted saharan dust events in LNLC environments: Impact on nutrient dynamics and primary production. Biogeosciences 11, 4783–4800 (2014).

[b16] Bonnet S., Guieu C., Chiaverini J., Ras J. & Stock A. Effect of atmospheric nutrients on the autotrophic communities in a low nutrient, low chlorophyll system. Limnol. Oceanogr. 50, 1810–1819 (2005).

[b17] Mills M. M., Ridame C., Davey M., La Roche J. & Geider R. J. Iron and phosphorus co-limit nitrogen fixation in the eastern tropical North Atlantic. Nature 429, 292–294 (2004). 15152251 10.1038/nature02550

[b18] Bonilla-Findji O., Gattuso J. P., Pizay M.-D. & Weinbauer M. G. Autotrophic and heterotrophic metabolism of microbial planktonic communities in an oligotrophic coastal marine ecosystem: Seasonal dynamics and episodic events. Biogeosciences 7, 3491–3503 (2010).

[b19] Lekunberri I. *et al.* Effects of a dust deposition event on coastal marine microbial abundance and activity, bacterial community structure and ecosystem function. J. Plankton Res. 32, 381–396 (2010).

[b20] Pulido-Villena E. *et al.* Microbial food web dynamics in response to a Saharan dust event: Results from a mesocosm study in the oligotrophic Mediterranean Sea. Biogeosciences 11, 5607–5619 (2014).

[b21] Ridame C. & Guieu C. Saharan input of phosphate to the oligotrophic water of the open western Mediterranean Sea. Limnol. Oceanogr. 47, 856–869 (2002).

[b22] Hoffmann L. J. *et al.* Influence of trace metal release from volcanic ash on growth of Thalassiosira pseudonana and Emiliania huxleyi. Mar. Chem. 132, 28–33 (2012).

[b23] Williamson C. E. *et al.* Solar ultraviolet radiation in a changing climate. Nat. Clim. Change 4, 434–441 (2014).

[b24] Ruiz-González C., Simó R., Sommaruga R. & Gasol J. M. Away from darkness: A review on the effects of solar radiation on heterotrophic bacterioplankton activity. Front. Microbiol. 4, 1–24 (2013). 23734148 10.3389/fmicb.2013.00131 PMC3661993

[b25] Helbling E. W., Gao K., Gonçalves R. J., Wu H. & Villafañe V. E. Utilization of solar UV radiation by coastal phytoplankton assemblages off SE China when exposed to fast mixing. Mar. Ecol. Prog. Ser. 259, 59–66 (2003).

[b26] Matallana-Surget S., Douki T., Cavicchioli R. & Joux F. Remarkable resistance to UVB of the marine bacterium *Photobacterium angustum* explained by an unexpected role of photolyase. Photochem. Photobiol. Sci. 8, 1313–1320 (2009). 19707619 10.1039/b902715g

[b27] Gao K. *et al.* Solar UV radiation drives CO_2_ fixation in marine phytoplankton: A double-edged sword. Plant Physiol. 144, 54–59 (2007). 17494919 10.1104/pp.107.098491 PMC1913777

[b28] Duarte C. M. & Prairie Y. M. Prevalence of heterotrophy and atmospheric CO_2_emissions from aquatic ecosystems. Ecosystems 8, 862–870 (2005).

[b29] Boyd P. W. *et al.* Physiological responses of a Southern Ocean diatom to complex future ocean conditions. Nat. Clim. Change 6, 207–213 (2016).

[b30] Brennan G. & Collins S. Growth responses of a green alga to multiple environmental drivers. Nat. Clim. Change 5, 892–897 (2015).

[b31] Hewitt J. E., Thrush S. F., Dayton P. K. & Bonsdorff E. The efect of spatial and temporal heterogeneity on the design and analysis of empirical studies of scale- dependent systems. Am. Nat. 169, 398–408 (2007). 17243075 10.1086/510925

[b32] Sandman A. N., Wikström S. A., Blomqvist M., Kautsky H. & Isaeus M. Scale-dependent influence of environmental variables on species distribution: a case study on five coastal benthic species in the Baltic Sea. Ecography 36, 354–364 (2013).

[b33] Häder D. P., Villafañe V. E. & Helbling E. W. Productivity of aquatic primary producers under global climate change. Photochem. Photobiol. Sci. 13, 1370–1392 (2014). 25191675 10.1039/c3pp50418b

[b34] Buma A. G. J. *et al.* Wavelength-dependent xanthophyll cycle activity in marine microalgae exposed to natural ultraviolet radiation. Eur. J. Phycol. 44, 515–524 (2009).

[b35] Zenoff V. F., Heredia J., Ferrero M., Siñeriz F. & Farías M. E. Diverse UV-B resistance of culturable bacterial community from high-altitude wetland water. Curr. Microbiol. 52, 359–362 (2006). 16604419 10.1007/s00284-005-0241-5

[b36] Agustí S., Regaudie-de-Gioux A., Arrieta J. M. & Duarte C. M. Consequences of UV-enhanced community respiration for plankton metabolic balance. Limnol. Oceanogr. 59, 223–232 (2014).

[b37] García-Corral L. S. *et al.* Ultraviolet radiation enhances Arctic net plankton community production. Geophys. Res. Lett. 41, 1–8 (2014).

[b38] Carrillo P. *et al.* Interactive effect of UVR and phosphorus on the coastal phytoplankton community of the Western Mediterranean Sea: Unravelling eco- physiological mechanisms. Plos One 10, e0142987 (2015). 26599583 10.1371/journal.pone.0142987 PMC4658109

[b39] Durán C., Medina-Sánchez J. M., Herrera G. & Carrillo P. Changes in the phytoplankton-bacteria coupling triggered by joint action of UVR, nutrients, and warming in Mediterranean high-mountain lakes. Limnol. Oceanogr. 61, 413–429 (2016).

[b40] Olsen Y. *et al.* A comparative study of responses in plankton food web structure and function in contrasting European coastal waters exposed to experimental nutrient addition. Limnol. Oceanogr. 51, 488–503 (2006).

[b41] Marañón E. *et al.* Degree of oligotrophy controls the response of microbial plankton to Saharan dust. Limnol. Oceanogr. 55, 2339–2352 (2010).

[b42] Agustí S. & Duarte C. M. Strong seasonality in phytoplankton cell lysis in the NW Mediterranean littoral. Limnol. Oceanogr. 45, 940–947 (2000).

[b43] Paytan A. *et al.* Toxicity of atmospheric aerosols on marine phytoplankton. Proc. Natl. Acad. Sci. 106, 4601–4605 (2009). 19273845 10.1073/pnas.0811486106 PMC2653564

[b44] Regaudie-de-Gioux A. & Duarte C. M. Patterns in planktonic metabolism in the Mediterranean Sea. Biogeosciences 6, 3081–3089 (2009).

[b45] García-Corral L. S., Martínez-Ayala J., Duarte C. M. & Agustí S. Experimental assessment of cumulative temperature and UV-B radiation effects on Mediterranean plankton metabolism. Front. Mar. Sci. 2, 48 (2015).

[b46] Agustí S. *et al.* Ubiquitous healthy diatoms in the deep sea confirm deep carbon injection by the biological pump. Nat. Commun. 6, 8 (2015). 10.1038/ncomms8608 PMC4510647 26158221

[b47] Duarte C. M. & Cebrián J. The fate of the marine autotrophic production. Limnol. Oceanogr. 41, 1758–1766 (1996).

[b48] Calbet A. Mesozooplankton grazing effect on primary production: A global comparative analysis in marine ecosystems. Limnol. Oceanogr. 46, 1824–1830 (2001).

[b49] Hernández-León S. & Ikeda T. A. global assessment of mesozooplankton respiration in the ocean. J. Plankton Res. 27, 153–158 (2005).

[b50] Acker J. G. & Leptoukh G. Online analysis enhances use of NASA Earth science data. Eos Trans. AGU 88, 14–17 (2007).

[b51] Morales-Baquero R., Pulido-Villena E. & Reche I. Atmospheric inputs of phosphorus and nitrogen to the southwest Mediterranean region: Biogeochemical responses of high mountain lakes. Limnol. Oceanogr. 51, 830–837 (2006).

[b52] Bullejos F. J., Carrillo P., Villar-Argaiz M. & Medina-Sánchez J. M. Roles of phosphorus and ultraviolet radiation in the strength of phytoplankton–zooplankton coupling in a Mediterranean high mountain lake. Limnol. Oceanogr. 55, 2549–2562 (2010).

[b53] Houpert L. *et al.* Seasonal cycle of the mixed layer, the seasonal thermocline and the upper-ocean heat storage rate in the Mediterranean Sea derived from observations. Prog. Oceanogr. 132, 333–352 (2015).

[b54] Pulido-Villena E., Wagener T. & Guieu C. Bacterial response to dust pulses in the western Mediterranean: Implications for carbon cycling in the oligotrophic ocean. Glob. Biogeochem. Cycles 22, GB1020 (2008).

[b55] Guieu C. *et al.* Large clean mesocosms and simulated dust deposition: A new methodology to investigate responses of marine oligotrophic ecosystems to atmospheric inputs. Biogeosciences 7, 2765–2784 (2010).

[b56] Vincent J. *et al.* Variability of mineral dust deposition in the western Mediterranean basin and South-East of France. Atmos. Chem. Phys. Discuss. 15, 34673–34717 (2015).

[b57] Guerzoni S., Molinaroli E. & Chester R. Saharan dust inputs to the western Mediterranean Sea: Depositional patterns, geochemistry and sedimentological implications. Deep Sea Res. Pt II 44, 631–654 (1997).

[b58] Jeffrey S. W. & Humphrey G. F. New spectrophotometric equations for determining chlorophylls a, b, c 1 and c 2 in higher plants, algae and natural phytoplankton. Biochem. Physiol. Pflanzen (BBP) 167, 191–194 (1975).

[b59] Hillebrand H., Dürselen C. D., Kirschtel D., Pollingher U. & Zohary T. Biovolume calculation for pelagic and benthic microalgae. J. Phycol. 35, 403–424 (1999).

[b60] Ribés M., Coma R. & Maria-Gili M. Seasonal variation of particulate organic carbon, dissolved organic carbon and the contribution of microbial communities to live particulate organic carbon in a shallow near-bottom ecosystem at the Northwestern Mediterranean Sea. J. Plankton Res. 21, 1077–1100 (1999).

[b61] Verity P. G. *et al.* Relationships between cell volume and the carbon and nitrogen content of marine photosynthetic nanoplankton. Limnol. Oceanogr. 37, 1434–1446 (1992).

[b62] Zubkov M. V., Burkill P. H. & Topping J. N. Flow cytometric enumeration of DNA-stained oceanic planktonic protists. J. Plankton Res. 29, 79–86 (2007).

[b63] Gasol J. M. & Del Giorgio P. Using flow cytometry for counting natural planktonic bacteria and understanding the structure of planktonic bacterial communities. Sci. Mar. 64, 197–224 (2000).

[b64] Zubkov M. V. & Burkill P. H. Syringe pumped high speed flow cytometry of oceanic phytoplankton. Cytom. A 69, 1010–1019 (2006). 10.1002/cyto.a.20332 16969799

[b65] Mercado J. M. *et al.* Diurnal changes in the bio-optical properties of the phytoplankton in the Alborán Sea (Mediterranean Sea). Estuar. Coastal S. Sci. 69, 459–470 (2006).

[b66] Posch T. *et al.* Precision of bacterioplankton biomass determination: a comparison of two fluorescent dyes, and of allometric and linear volume-to-carbon conversion factors. Aquat. Microb. Ecol. 25, 55–63 (2001).

[b67] Koroleff F. Simultaneous persulphate oxidation of phosphorus and nitogen compounds in water. In Report on the Baltic intercalibration workshop (ed Grasshoff K.) (Compiler, Kiel, Germany 1977).

[b68] Benner R. & Strom M. A critical evaluation of the analytical blank asociated with DOC measurements by high-temperature catalytic oxidation. Mar. Chem. 41, 153–160 (1993).

[b69] Steemann Nielsen E. The use of radio-active carbon (C^14^) for measuring organic production in the sea. J. Cons. Perm. Int. Explor. Mer. 18, 117–140 (1952).

[b70] APHA. Standard methods for the examination of water and wastewater. (American Public Health Association 1992).

[b71] del Giorgio P. A. & Cole J. J. Bacterial growth efficiency in natural aquatic systems. Ann. Rev. Ecol. System. 29, 503–541 (1998).

[b72] Schlitzer R. Ocean Data View. Alfred Wegener Institute for Polar and *Marine Research*, Bremerhaven, Germany. URL https://odv.awi.de/ (2015).

