# Supplementary material for: Saharan dust inputs and high UVR levels jointly alter the metabolic balance of marine oligotrophic ecosystems
Source: Sci Rep. 2016 Oct 24;6:35892. doi: 10.1038/srep35892 (PMC5075918; doi:10.1038/srep35892)
Supplement: Supplementary Information [file srep35892-s1.doc]

Supporting information for

**Saharan dust inputs and high UVR levels jointly alter the metabolic balance of marine oligotrophic ecosystems**

Marco J. Cabrerizo^*^, Juan Manuel Medina-Sánchez, Juan Manuel González-Olalla, Manuel Villar-Argaiz, Presentación Carrillo

*Corresponding author: Marco J. Cabrerizo, mjc@ugr.es

**Table S1**.- Mean (±SD) of the planktonic metabolic rates, total primary production (PP_total_) and community respiration (CR) (in mmol C m^-3^ d^-1^) and of the production (P) / respiration (R) ratio of the planktonic communities in the Southwestern Mediterranean Sea. The number of stations sampled and their trophic nature (autotrophic or heterotrophic), position (latitude and longitude), depth (in m), *in situ* temperature (T, ºC), the dissolved organic carbon (DOC, in µM), total phosphorus (TP, in µM) and nitrogen (TN, in µM) and chlorophyll *a* (Chl *a,* in µg L^-1^) concentrations in each station are shown. Note that samples in each station were taken from surface waters (5 m depth).

| Station | Position | Depth | *in situ* T | DOC | TP | TN | Chl *a* | PP_total_ | CR | P/R |
| --- | --- | --- | --- | --- | --- | --- | --- | --- | --- | --- |
| Autotrophic 1 | 35º59' N, 4º19' W | 284 | 16.84(0.77) | 213(31) | 0.09(0.03) | 357(0.30) | 1.90(0.30) | 36(2) | 33(12) | 1.09 |
| Autotrophic 2 | 36º32' N, 3º48' W | 113 | 21.58(1.37) | 193(45) | 0.60(0.04) | 313(69) | 0.68(0.12) | 55(1) | 40(0) | 1.38 |
| Autotrophic 3 | 36º21' N, 3º46' W | 430 | 20.15(1.66) | 323(176) | 0.48(0.08) | 309(14) | 2.24(1.47) | 66(8) | 43(12) | 1.53 |
| Autotrophic 4 | 36º09' N, 3º42' W | 289 | 18.05(0.60) | 229(80) | 0.65(0.09) | 256(11) | 1.47(0.42) | 64(8) | 46(12) | 1.39 |
| Autotrophic 5 | 36º32' N, 3º10' W | 200 | 20.45(1.20) | 189(70) | 0.50(0.04) | 297(21) | 1.50(0.93) | 81(24) | 81(19) | 1 |
| Autotrophic 6 | 36º25 N, 2º45' W | 995 | 22.06(0.76) | 119(10) | 0.24(0.03) | 292(5) | 0.63(0.04) | 79(16) | 43(11) | 1.84 |
| Autotrophic 7 | 36º27' N, 2º13' W | 180 | 23.18(0.41) | 123(8) | 0.50(0.03) | 248(33) | 0.37(0.00) | 118(1) | 96(2) | 1.23 |
| Autotrophic 8 | 36º08' N, 1º50' W | 830 | 23.64(0.03) | 150(56) | 0.50(0.04) | 228(17) | 0.47(0.09) | 113(0) | 105(0.1) | 1.08 |
| Heterotrophic 1 | 36º37' N, 4º24' W | 76 | 17.78(1.14) | 322(89) | 0.10(0.00) | 348(10) | 0.75(0.09) | 10(1) | 38(13) | 0.26 |
| Heterotrophic 2 | 36º21' N, 4º22' W | 740 | 22.19(0.95) | 281(12) | 0.80(0.11) | 307(10) | 0.80(0.21) | 32(18) | 86(27) | 0.37 |
| Heterotrophic 3 | 36º39' N, 2º25' W | 338 | 23.50(0.05) | 137(40) | 0.51(0.07) | 268(5) | 0.34(0.07) | 21(6) | 66(23) | 0.32 |
| Heterotrophic 4 | 36º40' N, 2º09' W | 87 | 23.65(0.30) | 177(80) | 0.45(0.08) | 237(5) | 0.47(0.00) | 21(0.1) | 63(16) | 0.33 |
| Heterotrophic 5 | 36º26' N, 1º55' W | 1438 | 23.75(0.66) | 147(15) | 0.38(0.03) | 272(4) | 0.50(0.05) | 36(1) | 86(19) | 0.42 |
| Heterotrophic 6 | 36º16' N, 1º43' W | 1973 | 23.69(0.04) | 162(17) | 0.78(0.13) | 319(49) | 0.47(0.06) | 11(4) | 74(32) | 0.15 |

**Table S2**.- Mean (±SD) of chlorophyll *a* (Chl *a,* in µg L^-1^), total phosphorus (TP, in µM), total nitrogen (TN, in µM), sestonic carbon (in µM), nitrogen (in µM) and phosphorus (in µM), sestonic C:P and N:P ratio and dissolved organic carbon concentrations (DOC, in µM) at the beginning of the experiment for the heterotrophic and autotrophic area.

|  | Heterotrophic area | Autotrophic area |
| --- | --- | --- |
| Chl *a* (µg L^-1^) | 0.75 (0.09) | 1.90 (0.30) |
| TP (µM) | 0.10 (0.00) | 0.09 (0.03) |
| TN (µM) | 348 (10) | 357 (0.30) |
| Sestonic C (µM) | 58 (6) | 34 (4) |
| Sestonic N (µM) | 8.49 (0.45) | 4.38 (0.47) |
| Sestonic P (µM) | 0.02 (0.00) | 0.02 (0.00) |
| Sestonic C:P ratio | 2915 (271) | 1681 (296) |
| Sestonic N:P ratio | 425 (19) | 219 (12) |
| DOC (µM) | 322 (89) | 213 (31) |

**Table S3**.- Results from two-way analysis of variance (ANOVA) for cell biomass (in mg C m^-3^) of autotrophic nanoplankton (ANP), autotrophic picoplankton (APP) and heterotrophic picoplankton (HPP) in the heterotrophic and autotrophic area with radiation, UVR (PAB, > 280 nm and P, > 400 nm) and dust (ambient [amb] and dust), as factors. *F* values represent *F-*test and p, represent p-values. Numbers in bold mean p < 0.05 and n.s. not significant differences.

|  | ANP | | APP | | HPP | |
| --- | --- | --- | --- | --- | --- | --- |
| Treatment | *F* | p | *F* | p | *F* | P |
| Heterotrophic | | | | | | |
| UVR | 0.15 | n.s. | 0.60 | n.s. | 0.29 | n.s. |
| Dust | 0.02 | n.s. | 5.34 | **< 0.05** | 1.22 | n.s. |
| UVR×Dust | 0.04 | n.s. | 1.46 | n.s. | 8.42 | **< 0.01** |
| Autotrophic | | | | | | |
| UVR | 71.27 | **< 0.001** | 3.81 | n.s. | 155.14 | **< 0.001** |
| Dust | 4.78 | n.s. | 0.01 | n.s. | 33.05 | **< 0.001** |
| UVR×Dust | 116.92 | **< 0.001** | 0.01 | n.s. | 6.43 | **< 0.05** |

**Table S4**.- Results from one-way repeated measures analysis of variance (RM-ANOVA) for the effect size of UVR on the total primary production (PP_total_) and community respiration (CR) in the heterotrophic and autotrophic area with dust (ambient [amb] and dust) and time, as factors. *F* values represent *F-*test and p, represent p-values. Numbers in bold mean p < 0.05 and n.s. not significant differences.

|  | PP_total_ | | CR | |
| --- | --- | --- | --- | --- |
| Treatment | *F* | p | *F* | p |
|  | Heterotrophic | | | |
| Dust | 4.13 | n.s. | 1.43 | n.s. |
| Time | 10.18 | **< 0.001** | 2.02 | n.s. |
| Dust×Time | 6.48 | **< 0.01** | 2.00 | **< 0.05** |
|  | Autotrophic | | | |
| Dust | 7.12 | **0.05** | 0.75 | n.s. |
| Time | 5.24 | **< 0.01** | 11.11 | **< 0.001** |
| Dust×Time | 15.16 | **< 0.001** | 13.69 | **< 0.001** |

**Figure S1.** (A) Daily area-average aerosol index (triangles, AI > 1, relative units), (B) inter-annual succession of AI events from 2012-2014 and (C) monthly area-average surface short-wave radiation fluxes (W m^-2^) on Southwestern Mediterranean Sea from 1979-2016 period.

**Figure S2**- Surface solar radiation for: (A) PAR, 400-700 nm (in W m^-2^) and (B) UV-B (305 nm, solid line) and UV-A (320 nm, large dashed line and 380 nm, small dashed line) (in µW cm^-2^) during the exposure period (June 17^th^-21^th^, 2014).

**Figure S3.-** (A, B) Mean (± SD) total primary production (PP_total_, in mmol C m^-3^ d^-1^) and (C, D) community respiration (CR) rates (in mmol C m^-3^ d^-1^) in the microcosms during the experiment under two radiation treatments, PAB (> 280 nm) and PAR (> 400 nm) and two dust treatments, ambient (amb) and dust in the heterotrophic and autotrophic area.

**Figure S4.-** Mean (± SD) total primary production / community respiration (PP_total_ / CR) ratios under two radiation treatments, PAB (> 280 nm) and PAR (P, > 400 nm) and two nutrient treatments, ambient (amb) and dust during the experiments in the heterotrophic and autotrophic area, considering the potential mesozooplankton grazing and respiration in unproductive marine waters,
